# Large-Scale Screening in General Population Children for Celiac Disease with a Multiplex Electrochemiluminescence (ECL) Assay

**DOI:** 10.1155/2020/8897656

**Published:** 2020-12-24

**Authors:** Ling He, Xiaofan Jia, Yong Gu, Dongmei Miao, Kathleen Waugh, Cristy Geno, Edwin Liu, Marian Rewers, Liping Yu

**Affiliations:** ^1^Barbara Davis Center for Diabetes, University of Colorado School of Medicine, Aurora, CO, USA; ^2^Department of Endocrinology, Guangzhou First People's Hospital, School of Medicine, South China University of Technology, Guangzhou, Guangdong, China; ^3^Department of Endocrinology, Beijing Hospital, National Center of Gerontology, Institute of Geriatric Medicine, Chinese Academy of Medical Sciences, China; ^4^University of Colorado Children's Hospital, Aurora, CO, USA

## Abstract

**Background:**

Autoimmunity Screening for Kids (ASK) study was launched to screen general population children for type 1 diabetes (T1D) and celiac disease (CD).

**Methods:**

A total of 23,319 children from general population were screened. A high throughput multiplex electrochemiluminescence (ECL) assay to screen multiautoantibodies in a single well was applied, parallel with a standard radiobinding assay (RBA). All children with any positive autoantibodies in screening were revisited within one month for confirmation and followed every 6 months.

**Results:**

Among 23,319 children, 2.6% (606/23,319) of children were tested positive for TGA. Multiplex ECL assay detected more TGA (584/23,319) in the initial screening than RBA (490/23,319, *p* = 0.004) and was able to detect TGA earlier than RBA in a subset of children by 0.8 to 34.8 months. Prevalence of TGA by either ECL or RBA in children with islet autoantibodies was found significantly higher than overall prevalence in general population screened.

**Conclusions:**

A multiplex ECL assay was more sensitive than standard RBA by identifying more TGA positivity and detecting TGA earlier in general population screening. It also provides a high efficient tool with its unique advantage of multiplexing measurements to screen for multiple autoimmune diseases simultaneously in general population.

## 1. Introduction

Celiac disease (CD) is a chronic intestinal autoimmune enteropathy precipitated by exposure to dietary gluten in genetically predisposed individuals. Unfortunately, a large percentage of patients have been missed for diagnosis due to its atypical symptoms and lack of attention from patients and doctors. Screening in children revealed that 50 to 70% of those affected are asymptomatic [1, 2]. Population studies from diverse groups in the US suggested that CD may be underdiagnosed, the overall prevalence of CD among US people surveyed was 0.71%, and prevalence among non-Hispanic whites was 1.01% [3]. Extensive research based on serological screening has demonstrated that 0.5-1% of the EU population suffers from undiagnosed CD and the pooled global prevalence of CD was 1.4% among 275,818 individuals [4]. Untreated CD increases the risk of many complications such as malignancy, osteoporosis, infertility, low birth-weight babies, and depression. Diagnostic delay has various adverse effects on the growth and development of children. It is of great significance to diagnose patients with small intestine enteropathy as early as possible before signs and symptoms of the disease develop [5]. Both large clinical trials for type 1 diabetes (T1D) including the Diabetes Autoimmunity Study in the Young (DAISY) and The Environmental Determinants of Diabetes in the Young (TEDDY) have been screening for CD in young children as the secondary endpoint [6, 7].

It has been recognized that transglutaminase autoantibodies (TGA) were specific for CD [8–10]. A workshop with international collaborative effort aiming at improving and standardizing TGA measurement was organized in 2009 [11] and found that the measurement using RBA was more quantitative and sensitive than traditional ELISA in detecting low-titer sera. The method of RBA for TGA has currently been used as a standard assay in many national/international clinical trials, but it has not been widely accepted because of its relying on radioactivity and cannot be performed by many laboratories. Recently, we have developed a nonradioactive TGA assay using electrochemiluminescence (ECL) detection [12]. The ECL assay has been demonstrated even more sensitive than current standard RBA and can detect TGA earlier in young children followed from the birth. A large set of patients with one autoimmune disease are accompanied with additional autoimmune conditions, and it is essential to identify these diseases simultaneously in screening for patients to achieve a better clinical care. Unfortunately, there is no easy and inexpensive tool to screen for these conditions. With the platform of our established ECL assay, we successfully developed a multiplex ECL assay [13] to determine multiautoantibodies simultaneously in one single well for multiple autoimmune diseases. In the present study, we applied our multiplex ECL assay in an ongoing clinical trial, the Autoimmunity Screening for Kids (ASK) [14], to screen for both CD and T1D in children from general population in Denver metropolitan area. The tests for TGA of CD and multiple islet autoantibodies of T1D were multiplexed in a single well with a multiplex ECL assay, and in parallel, the standard single RBAs were performed. The assay sensitivity, specificity, and stability of TGA in a multiplex ECL assay were analyzed and compared with the standard RBA.

## 2. Research Design and Methods

### 2.1. Subjects

A total of 23,400 children from general population in Denver metropolitan area were enrolled in the ASK study from December of 2016 to December of 2019, and 23,319 participant children had the TGA tested by both RBA and multiplex ECL assay. All children with any positive autoantibodies in screening were revisited within one month for confirmation and followed every 6 months. Signed written informed consents were obtained from participants, and the protocol was approved by the Colorado Multiple Institutional Review Board.

### 2.2. Multiplex ECL Assay

**Figure 1:**
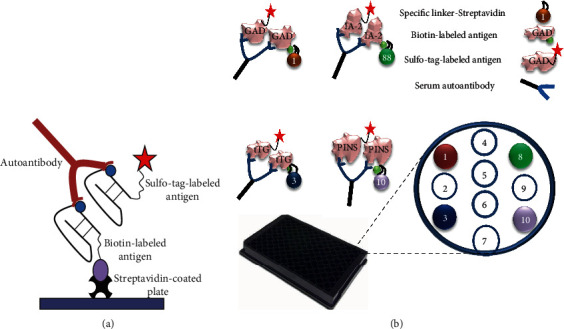
Illustration of ECL assay. (a) Single autoantibody ECL assay. The autoantibodies in serum bridge the sulfo-tagged antigen to the biotinylated antigen which will be captured by the streptavidin-coated plate. Detection of plate captured sulfo-tagged antigen is accomplished with ECL. (b) Multiplex autoantibody ECL assay. The autoantibodies in serum bridge the sulfo-tagged antigen to the biotinylated antigen which is coupled with a streptavidin-attached specific linker. Each specific linker coupled antibody-antigen complex will be limited in a designated spot area of each well. Detection of plate captured sulfo-tagged antigens is accomplished with ECL.

TGA screening was performed in a multiplex ECL assay format combined with other three islet autoantibodies, and all positive results were retested for confirmation using corresponding single ECL assays. The method of multiplex ECL assay has been published previously [13]. The mechanisms of both single and multiplex ECL assays are shown in Figure 1. In brief, 15 *μ*l of serum was mixed with 18 *μ*l of 0.5 M of acetic acid, incubated for 45 minutes at room temperature (RT); then, 25 *μ*l of acid-treated serum was transferred to a new well containing 35 *μ*l of freshly prepared antigen and neutralized with 13 *μ*l of 1 M Tris-HCl (pH = 9.0). The mixture was incubated at RT for 1 hour followed by incubation at 4°C overnight. Apply overnight incubates onto an ECL multiplex plate, incubate at RT for 1 hour, wash the plate, and then count it on a MSD Sector Imager SQ120 (MSD, Rockville, MD). The results were expressed as an index (index = [Signal_sample_–Signal_NegativeControl_]/[Signal_PositiveControl_–Signal_NegativeControl_]). The cut-off of 0.015 for ECL-TGA in a multiplex ECL assay was established on the 99th percentile of 1,022 healthy controls, and the interassay coefficiency of variation was 12.2% (*n* = 25).

### 2.3. Single ECL-TGA Assay

The method of ECL-TGA assay was previously published [12]. In brief, 20 *μ*l of 10x diluted serum with PBS was incubated with 20 *μ*l of antigen buffer containing both biotin and sulfo-tag-labeled transglutaminase protein for 1 hour at RT followed by incubation at 4°C overnight. On the 2nd day, 30 *μ*l of overnight incubates was transferred onto a streptavidin-coated plate (MSD, Gaithersburg, MD), incubated at RT for 1 hour. The plate was then washed and counted on a MSD Sector Imager SQ120 (MSD, Gaithersburg, MD). The result was expressed as an index (index = [Signal_sample_–Signal_NegativeControl_]/[Signal_PositiveControl_–Signal_NegativeControl_]). ECL-TGA cut-off of 0.015 was based on the 99th percentile of 1,022 healthy controls, and interassay coefficiency of variation was 8.1% (*n* = 30).

### 2.4. RBA-TGA

The method of RBA-TGA was published previously [15], and the upper limit of normal (index 0.050) was established as the 99th percentile of 1,022 healthy control subjects. The interassay CV for the RBA-TGA assay was 8.9% (*n* = 25).

### 2.5. Statistics

Regression line analysis was applied for the correlation of autoantibody levels between two assay methods. The rank-sum unpaired test was analyzed to compare the means for ages, levels of autoantibodies between groups. The difference between classification data was tested by the chi-squared test. Fisher's exact test was used for the prevalence of autoantibody positivity. All statistical tests were performed with PRISM 4.0 version software (GraphPad Software Inc., San Diego, CA), and a two-tailed *p* value with an alpha level for significance was set at 0.05.

## 3. Results

### 3.1. ECL Assay Was More Sensitive than RBA in Screening of General Population Children

**Figure 2:**
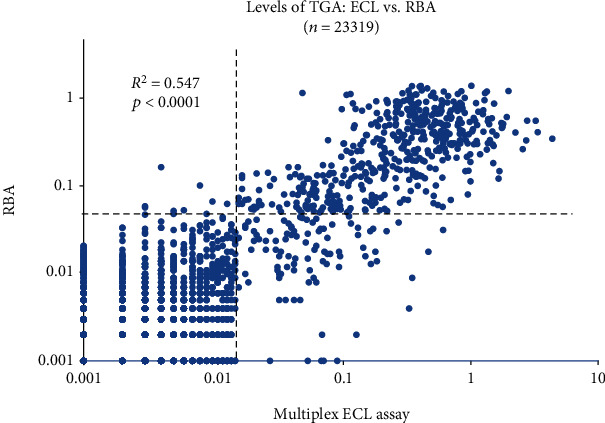
Levels of TGA: ECL vs. RBA on 23,319 children studied. ECL-TGA were compared with RBA-TGA in levels. The dotted lines represent the cut-offs for ECL-TGA and RBA-TGA, respectively. The levels of TGA from two assays were well correlated (*R*^2^ = 0.547, *p* < 0.0001). Significantly more TGA were detected by ECL than RBA.

Among 23,319 initial screening samples with TGA data available by both RBA and multiplex ECL assay, 606 children (2.6%) in total were tested TGA positive by multiplex ECL and/or RBA. Of 606 TGA positives, 468 were positive by both ECL and RBA, 116 positive by ECL only, and 22 positive by RBA only. With identical assay specificity set on 99th percentile in both assays, ECL assay detected significantly more TGA (2.50%, 584/23,319) than RBA (2.10%, 490/23,319, *p* = 0.004). The demographic data and TGA positivity in a multiplex ECL assay and RBA are summarized in Table 1. Age and female percentage were significantly higher in TGA-positive children than in TGA-negative children. The levels of TGA in children who were detected by both ECL assay and RBA were significantly higher in both assays: 0.317 (0.016-4.351) for ECL; 0.302 (0.051-1.458) for RBA than in children who were TGA positive by either ECL assay only: 0.054 (0.016-0.618), *p* < 0.01, or RBA only: 0.068 (0.053-0.245), *p* < 0.01. The absolute levels for both RBA and ECL assay were not discriminatory as illustrated in Figure 2. The levels of TGA between multiplex ECL assay and RBA were well correlated (*R*^2^ = 0.547, *p* < 0.0001).

### 3.2. ECL Assay Detected TGA Earlier than RBA in Longitudinally Followed Up Children

**Figure 3:**
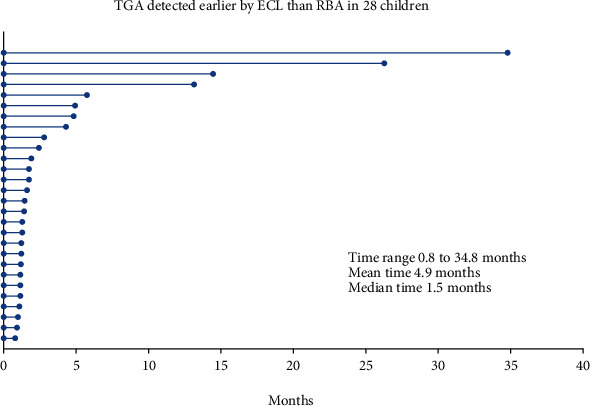
TGA were detected earlier in months in 28 children with multiplex ECL assay than RBA. The beginning of each line is TGA detecting time as the baseline of time with ECL assay for a child and the end of line is TGA detecting time with RBA.

Of all 606 TGA-positive subjects, 377 had available data on TGA follow-up for both RBA and ECL assay including 287 positives in both ECL and RBA, 75 ECL positive only, and 15 RBA positive only, and our study mainly aimed at observing TGA status change during the follow-up. The follow-up times so far were ranged from 0.5 to 34.8 months, mean 3.2 months, and median 1.5 months. Among 287 TGA positive by both RBA and ECL assay, 97.2% (279/287) of subjects were consistent, remained positive by both assays, and only 7 became negative. Among 75 TGA positive by ECL only, 82.7% (62/75) were persisted positive in which 28 RBA negative at initial screening were converted to RBA positive. In 15 TGA positive by RBA alone, most (*n* = 9) became TGA negative, a significantly higher rate of losing positivity than those by ECL assay alone (9/15, *p* = 0.001), and only 6 remained RBA positive in which 3 were found ECL positive in confirmation visits of one month later. Of 28 children who were detected TGA earlier by ECL than RBA, the times of earlier detection by ECL assay were ranged from 0.8 to 34.8 months (mean of 4.9 months; median 1.5 months) as illustrated in Figure 3.

### 3.3. TGA Positivity by ECL and/or RBA Is Associated with Islet Autoantibodies

**Figure 4:**
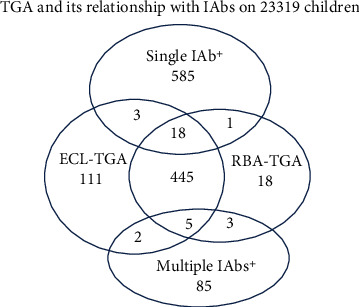
TGA positivity by ECL and RBA and its relationship with islet autoantibodies (IAbs) on 23,319 children screened in general population. Only small proportions of TGA (32/606, 5.3%) and IAbs (32/702, 4.6%) were overlapped between. The overlap of TGA with multiple IAbs was significantly higher than that with single IAb (10.5% (10/95) vs. 3.6% (22/607), *p* = 0.006).

The association of TGA positivity by both ECL and RBA with islet autoantibodies of T1D autoimmunity was analyzed. Compared with 2.6% (606/23319) of TGA prevalence by ECL and/or RBA in a total of 23,319 children screened, TGA prevalence in children who were detected positive for islet autoantibodies was found significantly higher (4.6% (32/702), *p* = 0.003). It is also true for the prevalence of ECL-TGA and RBA-TGA, respectively, 2.5% (584/23319) vs. 4.0% (28/702, *p* = 0.02) for ECL-TGA and 2.1% (490/23319) vs. 3.8% (27/702, *p* = 0.003) for RBA-TGA. Further analysis revealed that the prevalence of TGA in children who were positive for multiple IAbs was significantly higher than that in children who were single IAb positive, 10.5% (10/95) vs. 3.6% (22/607, *p* = 0.006). The relationship of TGA positivity either by ECL or by RBA with multiple IAbs or single IAb positivity is plotted in Figure 4.

## 4. Discussion

CD is recognized as a multifactorial autoimmune disorder with a variety of presentations [16]. The prevalence of CD is far higher than we recognized, besides its atypical symptoms or no apparent symptoms. Previous studies found that the complaining of CD-associated symptoms from patients was unrelated to clinical disease [2]. In another word, the frequency of complaining for CD-associated symptoms was identical between identified patients with CD and subjects without CD. It is difficult to recognize the CD according to the clinical symptoms, and thus, population-based screening of TGA will be essential to identify the disease early. One of current major barriers for large-scale population screening is lack of efficient screening methods. The ASK study is a ground-break to take an effort of mass CD screening in the general population for the first time in the United States [14]. The multiplex ECL assay combining multiple autoantibody assays in one single well with high sensitivity and specificity has its unique advantages of high efficiency and low cost to screen for multiple autoimmune diseases simultaneously in such a large-scale screening.

Our previous study [12] has demonstrated that ECL assay was more sensitive in detecting TGA in both cohorts, newly diagnosed patients with T1D, and nondiabetic young children followed from birth in DAISY. The results in the present study using a multiplex ECL assay in general population screening were consistent with our previous finding that ECL assay for TGA was more sensitive than currently standard RBA. ECL assay is unique in its ability to detect autoantibodies of all immunoglobulin classes including IgA, IgG, and IgM [17], which is helpful in individuals with selective IgA deficiency and early detection on the initial stage of antibody development with IgM only at seroconversion. Of 606 cases with positive TGA, near 20% (116/606) were positive by ECL assay alone whereas many of these cases were seen converted to RBA-TGA positive from initial negatives during the follow-up. This earlier detection of TGA using a multiplex ECL assay was consistent with our previous finding with a single ECL assay in the DAISY cohort of young children followed from birth. We believe, with the longer time of follow-up, more cases of children will be identified for the earlier detection of TGA by ECL assay. Earlier detections of autoantibodies by ECL assay were reported for other autoimmune diseases like T1D in our previous studies [18]. Autoantibodies to insulin (IAA) and GAD65 (GADA) are found as two earliest autoantibodies on the initial stage of T1D autoimmunity whereas ECL assays were shown to detect both IAA and GADA earlier than current standard RBA in both DAISY [18] and TEDDY (unpublished data) cohorts of young children followed from the birth. Both DAISY and TEDDY studies are aimed at identifying environmental factors responsible for triggering autoimmunity for T1D or CD. The time of initial autoantibody seroconversion is an essential checkpoint in both clinical trials, and early detection of autoantibodies with a more sensitive assay will pin-point more precisely the time of beginning of the autoimmune process.

In the present study of general population screening, we found the frequency of TGA for CD autoimmunity was significantly higher in children who were detected positive for islet autoantibodies of T1D autoimmunity (*p* = 0.003), compared with the overall prevalence of TGA in general population screened. Further analysis of TGA positivity among the children with positive islet autoantibodies revealed that the incidence of TGA was significantly more concentrated in children who had multiple islet autoantibodies and were at high risk for T1D (*p* = 0.006), compared with that in children who were positive only for single islet autoantibody and were at low risk. This is consistent with the previous findings that patients with one autoimmune disease are more likely to have other autoimmune conditions [19]. While compared with overall positivity, only small proportions of total TGA positivity (5.3%) and of total IAbs (4.6%) were overlapped with each other, which illustrated that CD and T1D are two independent autoimmune diseases.

This is an ongoing study, and the number of TGA screening positive cases have more coming. Many cases with positive TGA still have a short time or are absent yet for follow-up data, and the full spectrum of follow-up will not be known for the next few years. Finally, the study has a limitation of resources to provide complete clinical evaluation of intestinal biopsy for the confirmed positive cases whereas most of these children have been arranged for further clinical evaluation in their primary or outside care facilities.

In conclusion, a multiplex ECL assay for TGA was successfully applied in the study of mass screening of children from general population. ECL assay was more sensitive compared with current standard RBA and was able to identify more TGA positivity and to detect TGA earlier in a subset of children. This is the first general population-based study of mass screening for CD in the United States. The present study demonstrated a multiplex ECL assay in its unique advantage of multiplexing autoantibody measurements for multiple autoimmune diseases simultaneously in large-scale screening of general population.

## Figures and Tables

**Table 1:** Demographic data and TGA levels of the children screened.

	TGA+			TGA-
	ECL+ and RBA+	ECL+ only	RBA+ only	ECL- and RBA-
*N*	468	116	22	22713
Age (yr)	10.3 (2.0-17.9)^∗∗^	9.6 (2.1-17.9)	9.0 (1.9-17.5)	9.2 (0.1-17.9)
Sex (% female)	61%^∗^	59%	77%	51%
Level (ECL)	0.317 (0.016-4.351)^#^	0.054 (0.016-0.618)	-0.003 (-0.033-0.013)	-0.003 (-0.200-0.015)
Level (RBA)	0.302 (0.051-1.458)*^Δ^*	0.016 (-0.028-0.046)	0.068 (0.053-0.245)	-0.005 (-0.054-0.05)

vs. TGA- ^∗^*p* < 0.05, ^∗∗^*p* < 0.01; vs. ECL+ only ^#^*p* < 0.01; vs. RBA+ only *^Δ^p* < 0.01.

## Data Availability

The screening and following up data in general population children for celiac disease and type 1 diabetes used to support the findings of this study are available from the corresponding author upon request: liping.yu@cuanschutz.edu.

## Abbreviations

ECL: Electrochemiluminescence
RBA: Radiobinding assay
TGA: Transglutaminase autoantibodies
CD: Celiac disease
T1D: Type 1 diabetes
DAISY: Diabetes Autoimmunity Study in the Young
TEDDY: The Environmental Determinants of Diabetes in the Young
ASK: Autoimmunity Screening for Kids
RT: Room temperature.

## References

[1] Agardh D., Lee H. S., Kurppa K. (2015). Clinical features of celiac disease: a prospective birth cohort. *Pediatrics*.

[2] Rosen A., Sandstrom O., Carlsson A. (2014). Usefulness of symptoms to screen for celiac disease. *Pediatrics*.

[3] Rubio-Tapia A., Ludvigsson J. F., Brantner T. L., Murray J. A., Everhart J. E. (2012). The prevalence of celiac disease in the United States. *American Journal of Gastroenterology*.

[4] Singh P., Arora A., Strand T. A. (2018). Global prevalence of celiac disease: systematic review and meta-analysis. *Clinical Gastroenterology and Hepatology*.

[5] Murray J. A., Frey M. R., Oliva-Hemker M. (2018). Celiac disease. *Gastroenterology*.

[6] Lund-Blix N. A., Dong F., Mårild K. (2019). Gluten intake and risk of islet autoimmunity and progression to type 1 diabetes in children at increased risk of the disease: the Diabetes Autoimmunity Study in the Young (DAISY). *Diabetes Care*.

[7] Aronsson C. A., Lee H.-S., Hård Af Segerstad E. M. (2019). Association of gluten intake during the first 5 years of life with incidence of celiac disease autoimmunity and celiac disease among children at increased risk. *JAMA*.

[8] Martucciello S., Paolella G., Esposito C., Lepretti M., Caputo I. (2018). Anti-type 2 transglutaminase antibodies as modulators of type 2 transglutaminase functions: a possible pathological role in celiac disease. *Cellular and Molecular Life Sciences*.

[9] Dieterich W., Laag E., Schöpper H. (1998). Autoantibodies to tissue transglutaminase as predictors of celiac disease. *Gastroenterology*.

[10] Liu E., Bao F., Barriga K. (2003). Fluctuating transglutaminase autoantibodies are related to histologic features of celiac disease. *Clinical Gastroenterology and Hepatology*.

[11] Li M., Yu L., Tiberti C. (2009). A report on the international transglutaminase autoantibody workshop for celiac disease. *The American Journal of Gastroenterology*.

[12] Zhao Z., Miao D., Waugh K. (2016). Higher sensitivity and earlier identification of celiac disease autoimmunity by a nonradioactive assay for transglutaminase autoantibodies. *Journal of Immunology Research*.

[13] Gu Y., Zhao Z., Waugh K. (2019). High-throughput multiplexed autoantibody detection to screen type 1 diabetes and multiple autoimmune diseases simultaneously. *eBioMedicine*.

[14] Stahl M. G., Rasmussen G., Dong F. (2020). Mass screening for celiac disease: the Autoimmunity Screening for Kids (ASK) study. *The American Journal of Gastroenterology*.

[15] Bao F., Yu L., Babu S. (1999). One third of HLA DQ2 homozygous patients with type 1 diabetes express celiac disease-associated transglutaminase autoantibodies. *Journal of Autoimmunity*.

[16] Meis M., Adamiak T. (2018). Pediatric celiac disease - a review. *South Dakota Medicine*.

[17] Yu L. (2016). Islet autoantibody detection by electrochemiluminescence (ECL) assay. *Methods in Molecular Biology*.

[18] Yu L., Dong F., Miao D., Fouts A. R., Wenzlau J. M., Steck A. K. (2013). Proinsulin/insulin autoantibodies measured with electrochemiluminescent assay are the earliest indicator of prediabetic islet autoimmunity. *Diabetes Care*.

[19] Kakleas K., Soldatou A., Karachaliou F., Karavanaki K. (2015). Associated autoimmune diseases in children and adolescents with type 1 diabetes mellitus (T1DM). *Autoimmunity Reviews*.

